# Growth suppression in ADHD patients taking stimulants.

**DOI:** 10.1192/j.eurpsy.2026.11527

**Published:** 2026-07-31

**Authors:** T. Gutierrez Higueras

**Affiliations:** Child and Adolescent Psychiatry, Servicio Andaluz de Salud, Jaén, Spain

## Abstract

**Introduction:**

Attention deficit hyperactivity Disorder (ADHD) is one of the most commonly diagnosed childhood behavioral disorders, with an estimated prevalence of 7% among children and adolescents in Spain. First-line medical treatments include stimulants (methylphenidate and amphetamine) due to their effectiveness in controlling symptoms. Stimulants are generally safe but may cause temporary growth suppression during the first years of treatment, primarily due to loss of appetite and secondary reduction in caloric intake, as well as possible alterations in growth hormone secretion. However, the impact of their long-term use on final adult height remains uncertain.

**Objectives:**

To evaluate the impact of stimulant treatment on final height in children and adolescents with ADHD, considering the duration and consistency of treatment.

**Methods:**

A review of the literature was conducted in Pubmed using the keywords: “Stimulant”, “ADHD” and “height”. Studies describing final adult height in children and adolescents with ADHD between 6 and 17 years of age were included.

**Results:**

The reviewed studies show that children treated with stimulants could experience growth deceleration during the active treatment period. In most studies, no significant differences in final adult height were observed compared with controls. Other studies suggest that long-term stimulant use may be associated with a small but clinically relevant reduction in adult height. Growth trajectories indicate that growth suppression is often more pronounced in those receiving high doses and prolonged treatment, whereas cessation of the drug allows for catch-up growth.

**Conclusions:**

The use of stimulants in adolescents with ADHD can cause temporary growth suppression, which can usually be compensated for after discontinuation of treatment. However, it is unknown whether prolonged use from early childhood through adulthood could clinically affect final adult height.

**Disclosure of Interest:**

None Declared

